# Analysis, pretreatment and enzymatic saccharification of different fractions of Scots pine

**DOI:** 10.1186/1472-6750-14-20

**Published:** 2014-03-19

**Authors:** Monica Normark, Sandra Winestrand, Torbjörn A Lestander, Leif J Jönsson

**Affiliations:** 1 Department of Chemistry, Umeå University, Umeå SE-901 87, Sweden; 2 Department of Forest Biomaterials and Technology, Swedish University of Agricultural Sciences, Umeå SE-901 83, Sweden

**Keywords:** Scots pine, Chemical composition, Dilute-acid pretreatment, Enzymatic saccharification

## Abstract

**Background:**

Forestry residues consisting of softwood are a major lignocellulosic resource for production of liquid biofuels. Scots pine, a commercially important forest tree, was fractionated into seven fractions of chips: juvenile heartwood, mature heartwood, juvenile sapwood, mature sapwood, bark, top parts, and knotwood. The different fractions were characterized analytically with regard to chemical composition and susceptibility to dilute-acid pretreatment and enzymatic saccharification.

**Results:**

All fractions were characterized by a high glucan content (38-43%) and a high content of other carbohydrates (11-14% mannan, 2-4% galactan) that generate easily convertible hexose sugars, and by a low content of inorganic material (0.2-0.9% ash). The lignin content was relatively uniform (27-32%) and the syringyl-guaiacyl ratio of the different fractions were within the range 0.021-0.025. The knotwood had a high content of extractives (9%) compared to the other fractions. The effects of pretreatment and enzymatic saccharification were relatively similar, but without pretreatment the bark fraction was considerably more susceptible to enzymatic saccharification.

**Conclusions:**

Since sawn timber is a main product from softwood species such as Scots pine, it is an important issue whether different parts of the tree are equally suitable for bioconversion processes. The investigation shows that bioconversion of Scots pine is facilitated by that most of the different fractions exhibit relatively similar properties with regard to chemical composition and susceptibility to techniques used for bioconversion of woody biomass.

## Background

Lignocellulosic resources, such as wood, can be converted to fuels and chemicals that serve as renewable alternatives to commodities produced from fossil resources, such as petroleum [1,2]. Potential lignocellulosic feedstocks include residues from forestry and agriculture, as well as energy crops that are dedicated for production of energy carriers, and which include both woody and non-woody plants.

Since liquid fuels need to be produced in large quantities, there are good reasons to investigate utilization of a wide variety of lignocellulosic feedstocks and not only those that with relative ease can be converted by pretreatment, saccharification, and microbial fermentation. Boreal forests that cover large areas of the northern parts of the Palearctic and Nearctic regions mainly consist of coniferous trees. For instance, data for Sweden during the period 2006–2010 indicate that 82% of the forest resources (given as the standing volume) consisted of softwood [3]. In rough terms approx. one half of that was Norway spruce (*Picea abies*) and the other half was Scots pine (*Pinus sylvestris*). Softwood, including various species of pine, is also common in many other forests in temperate and subtropical areas of the world. Although softwood is typically recalcitrant with regard to biocatalytic conversion processes, there are also clear advantages with using softwood as feedstocks [4,5]. Softwood is typically rich in glucan, mannan, and galactan [6,7], which can be converted to hexose sugars that are generally preferred in comparison to pentose sugars by microbes producing cellulosic ethanol and other fermentation products. Furthermore, several countries have well established systems for sustainable management of coniferous forests, where the main product is high-quality sawn timber, but where a considerable fraction is left for other purposes as an abundant lignocellulosic resource that is available throughout the year.

Considerable efforts are currently devoted to investigate the recalcitrance of natural and genetically-engineered varieties of different plants that are intended to be used for saccharification and further on to production of fuels and chemicals by fermenting microbes [2]. With regard to softwoods that are primarily used for production of sawn timber, the varieties that are planted in managed forests today will provide the supply of lignocellulosic residues in the decades to come. Because of that, there are good reasons to investigate the recalcitrance of the softwood that is used today, as we can expect the same type of feedstock in the near future.

We have divided the lignocellulose of Scots pine, one of the most important commercial species of softwood, into seven different fractions. The seven fractions were analyzed with regard to chemical composition, susceptibility to thermochemical pretreatment, and susceptibility to enzymatic hydrolysis. Investigations in this area shed light upon the fundamental properties of the feedstock, and are relevant for the potential utilization of softwood for bioconversion to fuels and chemicals. In case differences between different fractions are discovered, there are also emerging possibilities to fractionate forest feedstocks in an industrial scale using advanced techniques [8]. Some of the fractions included in this study, such as bark and top parts, are nevertheless available today using conventional fractionation technology. Other fractions that were included in the study, such as juvenile and mature sapwood and heartwood, were of interest to compare mainly for fundamental reasons. Therefore, studies in this area are relevant both with regard to fundamental issues concerning bioconversion of lignocellulose, and for applications related to a transition towards a bio-based economy.

## Results and discussion

### Chemical composition of pine fractions

The chemical composition of fractions of Scots pine wood and bark was determined and the results are summarized in Table 1. The glucan content varied between 38 and 43%. The lowest value was found in knotwood, while heartwood exhibited slightly higher values (42-43%). All fractions had small amounts of arabinan (2%) and galactan (2-4%). The xylan content varied between 5 and 7%, and the mannan content was 10-14%. The total lignin content varied between 27 and 32%. Most of the lignin was Klason lignin, while a minor part was acid-soluble lignin (Table 1). The total lignin content of the sapwood (27-28%) was slightly lower than that of the heartwood (29-30%). This agrees with previous studies of softwood, such as Norway spruce [9]. The highest lignin content was determined for knotwood (32%) (Table 1). The extraction procedure was based on a 9:1 mixture of petroleum ether and acetone rather than on ethanol, since inefficient extraction may lead to an overestimate of the lignin content due to precipitation of extractives with the lignin [10]. However, it is still possible that the lignin content of the extractive-rich knotwood (9% extractives, Table 1) is somewhat overestimated. This notion is supported by the fact that the total mass balance for knotwood ended up at 105%, while all other fractions had a mass balance between 92 and 100% (Table 1). The ash contents of the pine wood fractions were low (0.2-0.3%), while the bark fraction, which also contained phloem tissue and some wood, had a higher ash content (0.9%).

**Table 1:** **Chemical composition of fractions of Scots pine**^**a**^

**Contents in % (w/w)**											
***Fraction***	***Arabinan ± SD***	***Galactan ± SD***	***Glucan ± SD***	***Mannan ± SD***	***Xylan ± SD***	***Klason lignin ± SD***	***Acid soluble lignin ± SD***	***Total lignin ± SD***	***Extractives ± SD***	***Ash ± SD***	***Total***^***b***^
Juvenile heartwood	2.0 ± 0.1	3.1 ± 0.1	42.7 ± 3.9	11.8 ± 0.2	6.6 ± 0.1	27.9 ± 0.3	1.6 ± 0.1	29.5 ± 0.4	4.5 ± 0.5	0.2 ± 0.1	100
Mature heartwood	1.8 ± 0.1	3.0 ± 0.1	42.2 ± 5.2	12.1 ± 0.2	5.3 ± 0.1	27.7 ± 0.1	1.4 ± 0.1	29.1 ± 0.2	4.4 ± 0.6	0.2 ± 0.1	98
Juvenile sapwood	1.9 ± 0.1	2.8 ± 0.2	39.7 ± 0.1	10.5 ± 0.3	6.2 ± 0.3	25.8 ± 0.4	1.7 ± 0.1	27.4 ± 0.5	3.0 ± 0.2	0.3 ± 0.1	92
Mature sapwood	1.7 ± 0.1	2.3 ± 0.1	41.8 ± 1.6	14.0 ± 0.2	5.5 ± 0.1	26.9 ± 0.2	1.5 ± 0.1	28.4 ± 0.2	2.9 ± 0.1	0.2 ± 0.1	97
Bark	2.4 ± 0.2	2.8 ± 0.2	41.8 ± 2.0	11.7 ± 0.3	5.4 ± 0.1	28.2 ± 0.3	1.9 ± 0.1	30.0 ± 0.3	3.5 ± 0.2	0.9 ± 0.3	99
Top parts	2.0 ± 0.1	3.4 ± 0.1	41.4 ± 1.4	11.4 ± 0.1	6.6 ± 0.1	28.1 ± 0.2	1.6 ± 0.1	29.7 ± 0.2	3.3 ± 0.6	0.3 ± 0.1	98
Knotwood	2.2 ± 0.1	4.1 ± 0.3	38.2 ± 1.4	11.9 ± 0.1	6.6 ± 0.1	30.0 ± 0.3	1.6 ± 0.1	31.5 ± 0.3	9.2 ± 0.3	0.3 ± 0.1	105

^a^Yield in g 100 g^-1^ wood/bark. Mean values and standard deviations based on three replicates.

^b^Includes arabinan, galactan, glucan, mannan, xylan, total lignin, extractives, and ash.

As expected, the xylan content of the Scots pine fractions was low in relation to literature data for the xylan content of agricultural residues, such as sweet sorghum bagasse (17.2%), corn stover (18.9%), and wheat straw (18.4%) [6]. Prominent softwood hemicelluloses include galactoglucomannan and arabinoglucuronoxylan [7]. The total carbohydrate content of the Scots pine fractions varied between 61 and 66%, which could be compared with literature data for *Populus tremuloides* (58.3%), birchwood (57.9%), sugarcane bagasse (64.3%), wheat straw (57.1%), and corn stover (57.7%) (adapted from [6]). Sjöström [7] reported a lignin content of 27.7% for *P. sylvestris*, which corresponds well with the values of the present study. As expected, the lignin content of the pine fractions was higher than the lignin-content values reported for hardwood species, such as *Populus tremuloides* (16.7%) and birchwood (22.8%), and agricultural residues, such as sugarcane bagasse (18.6%) and wheat straw (17.6%) [6]. Knotwood is known to have a high content of extractives and attempts have been made to collect knotwood fractions for preparation of bioactive extracts [11]. Lestander et al. [12] reported ash contents of 0.3-0.4% in various types of wood (Scots pine, Norway spruce and birch), while the average ash content in the bark of Scots pine was 2.0%. The lower value of the ash content of bark reported in this study compared to the value reported by Lestander et al. [12] can be attributed to the presence of phloem tissue and some wood in the bark fraction of our study. The ash content of the pine fractions is very low compared to that of many agricultural residues considered for utilization in lignocellulosic biorefineries, for example sweet sorghum bagasse (4.2%), corn stover (4.9%), and wheat straw (5.8%) [6]. In a literature review by Tao et al. [13] covering 742 data objects it was shown that straw of herbaceous grasses contains in average more than 3% ash and up to about 18% in rice. Kenney et al. [14] have reported a typical ash content of 6-8% in a dataset of 840 samples from corn stover, Miscanthus and wheat straw. A high content of carbohydrates that can be hydrolyzed to hexose sugars and a low ash content are advantageous properties from a biorefining perspective.

**Figure 1:**
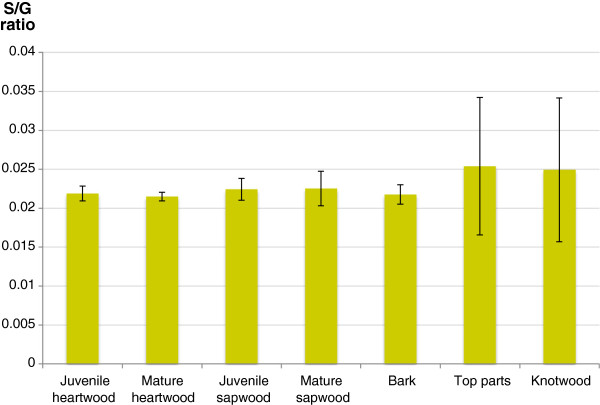
**Estimation of the syringyl/guaiacyl (S/G) ratios of different fractions of Scots pine based on analysis using Py-GC/MS.** The error bars show the standard deviations of six replicates.

Figure 1 shows the syringyl/guaiacyl (S/G) ratio of the different fractions from Scots pine. The determined S/G ratios varied between 0.021 and 0.025 but the variation between different fractions was not statistically significant (p > 0.05, Student’s t-test). The result indicates a very high proportion of guaiacyl residues in the lignin of all of the fractions. Lignin from conifers consists mainly of guaiacyl units derived from the monolignol coniferyl alcohol, whereas hardwood contain varying ratios of syringyl and guaiacyl units [15]. Glasser and Glasser [16] reported an S/G ratio for Loblolly pine of 0.023, which correlates well with the S/G ratios of the Scots pine fractions (Figure 1). The lignin content and the S/G ratio have been studied in relation to sugar release in enzymatic saccharification of different varieties of *Populus*[17,18]. A high lignin content has been associated with a low level of sugar release. Reports differ with respect to the potential influence of the S/G ratio on sugar release.

### Pretreatment and pretreatment liquids

Pretreatment was performed using sulfuric acid as catalyst, which is a suitable method for recalcitrant feedstocks such as softwood [5], and which is relevant for industrial-scale biorefining. The pretreatment was performed with varying severity: mild, medium and severe (Table 2). The resulting pretreatment liquids were analyzed with regard to the monosaccharide content using high-performance anion-exchange chromatography (HPAEC) (Table 3). Juvenile sapwood was used as the reference fraction in comparisons between the different fractions, due to that it was quantitatively dominating in this material. After mild pretreatment, the monosaccharide yields in the pretreatment liquid of all the different fractions followed the order glucose > mannose > galactose > arabinose, xylose. The total amount of sugar was higher in pretreatment liquids obtained after pretreatment with medium severity (Table 3). Except for the xylose yield of mature heartwood, the yields of all separate monosaccharides from all fractions were higher after pretreatment with medium severity than after pretreatment with mild severity. The pretreatment liquids obtained after pretreatment using severe conditions generally contained less sugar than the pretreatment liquids obtained after pretreatment using medium conditions (Table 3). However, the glucose yields from knotwood, juvenile heartwood and mature sapwood were slightly higher after severe pretreatment than after medium pretreatment. No xylose and very small amounts of arabinose could be detected in pretreatment liquids after severe pretreatment (Table 3), which indicates that the pentose sugars were sensitive to the harsh conditions and had been largely degraded to furfural and possibly further on to carboxylic acids [19].

**Table 2:** Different conditions used for pretreatment of fractions of Scots pine

	**Combined severity (log Ro-pH)**	**Temperature (°C)**	**Time (min)**	**H**_**2**_**SO**_**4**_**(% w/w)**^**a**^
Mild	2.7	180	6.0	2.0
Medium	3.0	180	6.0	4.0
Severe	3.3	180	12.0	4.0

^a^The loading (in percent) refers to the weight of sulfuric acid in relation to the total weight of the liquid added to the milled wood.

**Table 3:** **Sugar yields in pretreatment liquids**^**a**^

	**Pretreatment severity: mild**				
***Fraction***	***Arabinose***	***Galactose***	***Glucose***	***Mannose***	***Xylose***
Juvenile heartwood	0.1 ± 0.0	0.5 ± 0.1	3.7 ± 0.4	1.3 ± 0.3	0.1 ± 0.0
Mature heartwood	0.1 ± 0.0	0.4 ± 0.1	3.3 ± 0.3	1.3 ± 0.4	0.1 ± 0.0
Juvenile sapwood	0.1 ± 0.0	0.5 ± 0.1	3.8 ± 0.2	1.4 ± 0.3	0.1 ± 0.1
Mature sapwood	0.2 ± 0.1	0.4 ± 0.1	4.0 ± 0.3	1.9 ± 0.2	0.1 ± 0.0
Bark	0.3 ± 0.1	0.6 ± 0.1	3.9 ± 0.5	1.7 ± 0.5	0.1 ± 0.0
Top parts	0.2 ± 0.1	0.8 ± 0.1	3.5 ± 0.3	1.7 ± 0.2	0.2 ± 0.3
Knotwood	0.2 ± 0.1	1.0 ± 0.2	3.4 ± 0.3	1.6 ± 0.4	0.1 ± 0.0
	**Pretreatment severity: medium**				
***Fraction***	***Arabinose***	***Galactose***	***Glucose***	***Mannose***	***Xylose***
Juvenile heartwood	0.5 ± 0.1	1.8 ± 0.3	13.0 ± 1.0	4.8 ± 0.6	0.8 ± 0.1
Mature heartwood	n.d.^b^	0.8 ± 0.5	9.7 ± 1.2	2.6 ± 1.7	n.d.
Juvenile sapwood	0.6 ± 0.1	1.5 ± 0.1	13.3 ± 1.9	5.2 ± 0.2	0.9 ± 0.1
Mature sapwood	0.4 ± 0.1	0.8 ± 0.4	8.8 ± 2.5	3.9 ± 1.5	0.5 ± 0.2
Bark	0.9 ± 0.2	1.3 ± 0.3	13.7 ± 1.7	4.1 ± 0.9	0.6 ± 0.3
Top parts	0.6 ± 0.2	2.1 ± 0.6	12.1 ± 3.4	4.8 ± 1.2	0.8 ± 0.3
Knotwood	0.7 ± 0.1	2.5 ± 0.1	11.2 ± 1.0	5.3 ± 0.2	0.9 ± 0.1
	**Pretreatment severity: severe**				
***Fraction***	***Arabinose***	***Galactose***	***Glucose***	***Mannose***	***Xylose***
Juvenile heartwood	0.3 ± 0.1	1.0 ± 0.1	15.9 ± 2.0	1.9 ± 0.4	n.d.
Mature heartwood	n.d.	0.5 ± 0.2	9.4 ± 1.9	0.9 ± 0.5	n.d.
Juvenile sapwood	0.2 ± 0.1	0.6 ± 0.1	10.9 ± 1.6	n.d.	n.d.
Mature sapwood	n.d.	0.5 ± 0.1	8.9 ± 2.4	1.3 ± 0.3	n.d.
Bark	0.3 ± 0.1	0.5 ± 0.1	9.8 ± 0.7	0.5 ± 0.1	n.d.
Top parts	n.d.	0.8 ± 0.3	10.2 ± 1.7	0.8 ± 0.4	n.d.
Knotwood	0.4 ± 0.1	1.3 ± 0.3	13.1 ± 1.7	1.9 ± 0.6	n.d.

^a^Yield in g sugar 100 g^-1^ wood (dry weight) in samples taken after 72 h and analyzed using HPAEC. Mean values and standard deviations based on three replicates.

^b^Not detected: yield <0.001 g sugar 100 g^-1^ wood (values under the lowest standard, 0.5 ppm, in the calibration curve).

**Figure 2:**
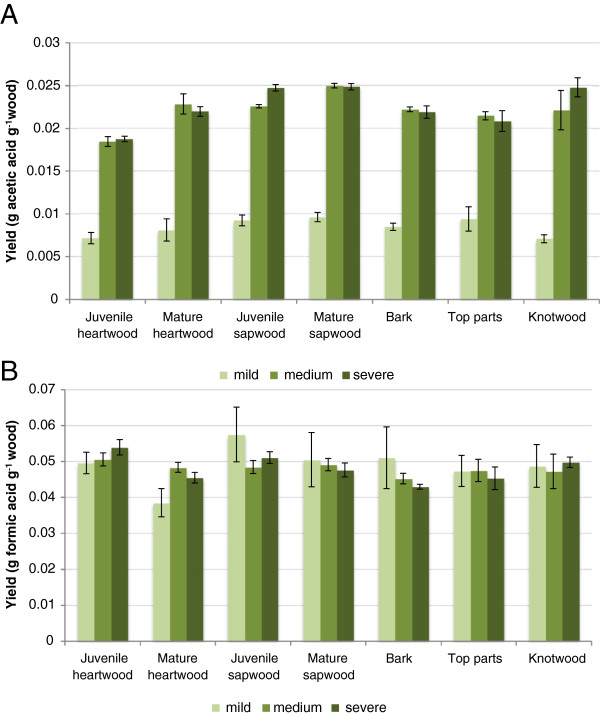
**Mean values of the yields of (A) acetic acid and (B) formic acid expressed as g acid g**^**-1 **^**wood in the pretreatment liquid of fractions of Scots pine.** The different pretreatment conditions are specified in Table 2. The error bars show the standard deviations of three replicates.

Some aliphatic acids, such as formic acid, are formed during pretreatment through thermochemical degradation of carbohydrates, while acetic acid is formed by hydrolysis of acetyl groups in xylan [19]. Low yields of both acetic acid and formic acid were obtained in the pretreatment liquid as an effect of the dilute-acid pretreatment (Figure 2). The differences between the yields of the acids from the different fractions were small (Figure 2). However, there was a very clear difference between the formation of acetic acid and formic acid in the sense that the concentrations of formic acid were rather similar regardless of whether the pretreatment conditions were mild, intermediate or severe, while the concentrations of acetic acid increased very much when the pretreatment changed from mild to intermediate conditions (Figure 2). When the pretreatment changed from intermediate to severe, there was no corresponding increase in the concentrations of acetic acid (Figure 2). This suggests that a combined severity (CS) of 3.0 (Table 2) was sufficient for quantitative hydrolysis of the acetyl groups in the different pine fractions.

The effects of aliphatic acids (acetic, formic and levulinic acids) on the ethanol yield of *S. cerevisiae* were studied by Larsson et al. [20] who found that acid concentrations over 100 mM tended to result in decreased ethanol yields, while concentrations lower than 100 mM tended to increase the ethanol yield. The pretreatment liquids of the different pine fractions showed very low concentrations of both acetic acid (<1.3 g/L, which correspond to approx. 25 mM) and formic acid (<2.8 g/L, which correspond to approx. 50 mM) for all pretreatment conditions. These low concentrations would not be expected to reach an inhibitory level but rather stimulate the ethanol yield in an ethanolic fermentation with yeast.

### Enzymatic saccharification

The susceptibility of untreated and pretreated pine fractions to enzymatic hydrolysis of cellulose was investigated using an analytical small-scale saccharification assay. Advantages with performing analytical saccharification in small scale include that large series of biomass samples can be processed in parallel, and that the number of replicates can be large enough to allow statistical analysis of the results.

**Figure 3:**
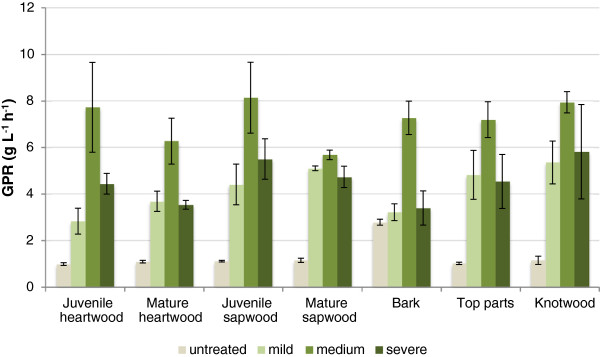
**Mean values of glucose production rates (GPR) after 4 h of enzymatic hydrolysis of fractions of Scots pine, with or without pretreatment (pretreatment conditions as specified in Table **2**).** The error bars show the standard deviations of three replicates.

Figure 3 shows the glucose production rates (GPR) after 4 h of hydrolysis of the seven pine fractions. Pretreatment always gave higher GPR values than hydrolysis without pretreatment. However, the bark fraction gave relatively high GPR even without pretreatment and the GPR did not improve significantly (p > 0.05, Student’s t-test) with mild pretreatment. It is noteworthy that the highest GPR values were always achieved using intermediate pretreatment conditions, and that increasing the CS to 3.3 did not give any further improvement. Compared to the reference fraction (juvenile sapwood), mild pretreatment resulted in statistically significantly (p < 0.05, Student’s t-test) lower GPR values for the juvenile heartwood and the bark fraction. No significant differences compared to the reference fraction could be detected for the intermediate pretreatment conditions. At severe pretreatment, significantly (p < 0.05, Student’s t-test) lower GPR compared to the reference could be detected for both the bark fraction and the mature heartwood.

Table 4 shows the sugar yields after enzymatic hydrolysis of the pine fractions, with or without pretreatment. The pretreated fractions gave none or very low yields of the pentose sugars arabinose and xylose. This is expected, as the pretreatment targets hemicelluloses. However, as the yields of arabinose and xylose in the pretreatment liquids (Table 3) were higher after medium pretreatment than after mild pretreatment, it would have been theoretically possible to obtain pentoses after enzymatic hydrolysis of fractions pretreated under mild conditions, especially since enzymatic hydrolysis of fractions that were not pretreated did generate some arabinose and xylose (Table 4). The fact that the enzymatic hydrolysates obtained from fractions that were not pretreated always contained all five monosaccharides analysed (Table 4) shows that the enzymes needed to generate these sugars were available in the enzyme preparations added. The enzymatic hydrolysates of fractions that had been pretreated using mild conditions contained galactose and mannose, while the fractions that were pretreated using medium or severe conditions did not (Table 4). As the highest yields of galactose and mannose in pretreatment liquids were achieved after pretreatment using medium conditions (Table 3), the fractions pretreated under medium and severe conditions were probably depleted of galactan and mannan, which explains that no or little galactose and mannose was generated in the enzymatic hydrolysis of the corresponding fractions. The glucose yield typically increased with increasing pretreatment severity (Table 4). For the bark fraction, the glucose yield was relatively high even without pretreatment and did not increase very much after pretreatment under mild or medium conditions (Table 4). Juvenile heartwood gave the lowest glucose yield without pretreatment or with mild pretreatment, but gave the highest glucose yield after severe pretreatment (Table 4). The medium pretreatment gave slightly higher glucose yields for sapwood fractions than for heartwood fractions, and the severe pretreatment gave slightly higher yields for juvenile fractions than for mature fractions, but these differences were not significant (p > 0.05, Student’s t-test).

**Table 4:** **Sugar yields after enzymatic hydrolysis**^**a**^

	**Untreated**				
***Fraction***	***Arabinose***	***Galactose***	***Glucose***	***Mannose***	***Xylose***
Juvenile heartwood	0.2 ± 0.1	0.4 ± 0.1	3.0 ± 0.3	0.7 ± 0.1	0.3 ± 0.1
Mature heartwood	0.2 ± 0.1	0.4 ± 0.1	3.3 ± 0.3	0.8 ± 0.1	0.3 ± 0.1
Juvenile sapwood	0.5 ± 0.1	0.7 ± 0.1	4.1 ± 0.2	0.9 ± 0.2	0.4 ± 0.2
Mature sapwood	0.2 ± 0.2	0.4 ± 0.2	3.3 ± 0.1	0.9 ± 0.2	0.3 ± 0.2
Bark	0.2 ± 0.1	0.4 ± 0.1	6.4 ± 0.6	0.8 ± 0.1	0.3 ± 0.1
Top parts	0.2 ± 0.1	0.4 ± 0.1	3.0 ± 0.3	0.7 ± 0.1	0.3 ± 0.1
Knotwood	0.2 ± 0.1	0.4 ± 0.1	3.3 ± 0.3	0.8 ± 0.1	0.3 ± 0.1
	**Pretreatment severity: mild**				
***Fraction***	***Arabinose***	***Galactose***	***Glucose***	***Mannose***	***Xylose***
Juvenile heartwood	n.d.^b^	0.58 ± 0.1	6.9 ± 2.6	0.7 ± 0.1	n.d.
Mature heartwood	n.d.	0.62 ± 0.1	8.4 ± 1.3	0.8 ± 0.1	n.d.
Juvenile sapwood	n.d.	0.65 ± 0.1	8.5 ± 1.0	0.8 ± 0.1	n.d.
Mature sapwood	n.d.	0.64 ± 0.1	10.6 ± 0.7	0.9 ± 0.2	n.d.
Bark	n.d.	0.63 ± 0.1	8.1 ± 1.3	0.8 ± 0.1	n.d.
Top parts	n.d.	0.62 ± 0.1	10.5 ± 2.3	0.8 ± 0.1	n.d.
Knotwood	n.d.	0.61 ± 0.1	10.6 ± 2.4	0.8 ± 0.1	n.d.
	**Pretreatment severity: medium**				
***Fraction***	***Arabinose***	***Galactose***	***Glucose***	***Mannose***	***Xylose***
Juvenile heartwood	n.d.	n.d.	10.1 ± 1.8	n.d.	n.d.
Mature heartwood	n.d.	n.d.	8.3 ± 0.7	n.d.	n.d.
Juvenile sapwood	n.d.	n.d.	12.3 ± 1.0	n.d.	n.d.
Mature sapwood	n.d.	n.d.	12.1 ± 2.4	n.d.	n.d.
Bark	n.d.	n.d.	7.5 ± 0.9	n.d.	n.d.
Top parts	n.d.	n.d.	9.2 ± 2.2	n.d.	n.d.
Knotwood	n.d.	n.d.	10.5 ± 1.4	n.d.	n.d.
	**Pretreatment severity: severe**				
***Fraction***	***Arabinose***	***Galactose***	***Glucose***	***Mannose***	***Xylose***
Juvenile heartwood	n.d.	n.d.	18.0 ± 1.4	n.d.	n.d.
Mature heartwood	n.d.	n.d.	15.8 ± 2.3	n.d.	n.d.
Juvenile sapwood	n.d.	n.d.	17.9 ± 2.5	n.d.	n.d.
Mature sapwood	n.d.	n.d.	14.6 ± 3.0	n.d.	n.d.
Bark	n.d.	n.d.	14.7 ± 0.3	n.d.	n.d.
Top parts	n.d.	n.d.	15.9 ± 2.0	n.d.	n.d.
Knotwood	n.d.	n.d.	16.6 ± 0.5	n.d.	n.d.

^a^Yield in g sugar 100 g^-1^ wood (dry weight) in samples taken after 72 h and analyzed using HPAEC. Mean values and standard deviations based on three replicates.

^b^Not detected: yield <0.001 g sugar 100 g^-1^ wood (values under the lowest standard, 0.5 ppm, in the calibration curve).

**Figure 4:**
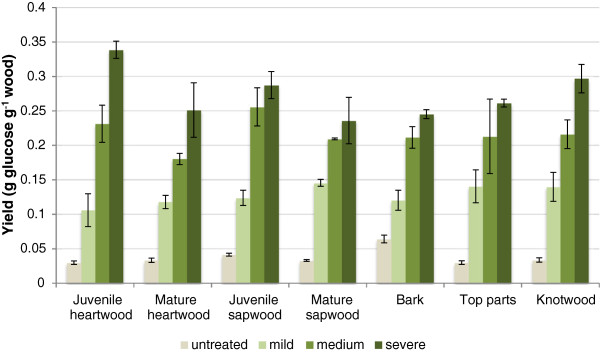
**The combined glucose yield in pretreatment liquids and enzymatic hydrolysates.** The figure shows mean values of glucose yields (expressed as g glucose per g wood after pretreatment and 72 h of enzymatic hydrolysis at 45°C in a orbital shaker set at 170 rpm). The error bars show the standard deviations of three replicate reactions for each pine fraction and pretreatment condition.

Figure 4 shows the combined glucose yield in pretreatment liquids and enzymatic hydrolysates after 72 h of hydrolysis. The yield without pretreatment was always much lower than the yield of pretreated pine wood and bark fractions. The glucose yield of all fractions increased with increasing severity of the pretreatment. There is a difference in that regard compared with the GPR values, which were based on samples taken after only 4 h of hydrolysis and for which the intermediate pretreatment gave higher values than those obtained for severe pretreatment. Without pretreatment all fractions showed significantly (p < 0.05, Student’s t-test) lower glucose yield compared to the reference fraction, except the bark fraction, which gave 55% higher glucose yield. A possible explanation for this is that the morphology of the bark is different from that of the wood and provides a more porous structure that improves the enzymatic accessibility during the hydrolysis. With mild pretreatment conditions a significant (p < 0.05, Student’s t-test) increase of glucose yield compared to the reference could be detected for the mature sapwood fraction. With medium pretreatment conditions, both mature sapwood and mature heartwood showed significantly lower glucose yield compared to the reference. With severe pretreatment conditions, the juvenile heartwood gave significantly higher glucose yield compared to the reference, whereas the bark fraction showed significantly lower yield.

Conversion of bark-rich softwood residues from hemlock using two-step acid hydrolysis was studied by Kim et al. [21], who found that using this approach it was possible to reach relatively high yields of hemicellulosic sugars, such as mannose, galactose, and xylose. Boussaid et al. [22] investigated steam pretreatment of wood chips of sapwood and heartwood of a 129-year-old Douglas fir. The sapwood fraction was found to be more susceptible to acid-catalyzed steam pretreatment and enzymatic hydrolysis at the medium severity level (195°C, 4.5% of SO_2_, 4.5 min). The way the wood was fractionated was different in that study [22] compared to our study, in which the wood was divided to a larger number of fractions including fractions with juvenile and mature wood. It is nevertheless possible to distinguish a similar trend for the Scots pine fractions with regard to the glucose yields (g glucose per g wood) from the total (juvenile and mature fractions combined) sapwood and heartwood fractions after pretreatment at mild and medium severity. After pretreatment under severe conditions, the heartwood fractions of Scots pine instead gave higher glucose yield (0.59 g glucose per g wood) than the sapwood fractions (0.52 g glucose per g wood).

The fact that pretreatment under severe conditions resulted in the highest glucose yields for all fractions (Table 4, samples taken after 72 h) while the highest GPR values were achieved with material pretreated at medium severity (Figure 3, samples taken after 4 h) suggests that the highest severity caused formation of something that retarded the initial cellulose hydrolysis rate. This effect cannot have been caused by sugars or other soluble compounds that would inhibit cellulases, since the pretreatment liquid, which contained the soluble compounds, was removed from the solid residue before the enzymatic hydrolysis. Acid-catalyzed dehydration of carbohydrates is responsible for formation of pseudo-lignin, an aromatic material with positive Klason lignin value that is derived from carbohydrates rather than from native lignin [23-27]. Pseudo-lignin is suggested to cause non-productive binding of enzymes, which will affect enzymatic hydrolysis in a negative way. It is possible that the highest severity resulted in formation of pseudo-lignin to an extent that slowed down the initial phase of the hydrolysis of cellulose, resulting in a lower GPR, but that it was possible for the enzymes to overcome this obstacle in the long run. Evidently, there was also a positive effect of the severe pretreatment compared to the intermediate pretreatment, since the glucose yields after 72 h were higher. This could have been due to incomplete removal of hemicelluloses for the intermediate pretreatment, but the data do not support that hypothesis since the concentrations of hemicellulosic sugars were not higher in pretreatment liquid from the severe pretreatment than in pretreatment liquid from the intermediate pretreatment (Table 3), and since the enzymatic treatment did not release any hemicellulosic sugars from the material pretreated at medium severity (Table 4). Increased severity reduces the degree of polymerization (DP) of cellulose which could improve the digestibility [28], but differences in the DP may be difficult to relate to the susceptibility to enzymatic hydrolysis [29]. Assuming limited accessibility to cellulose to be the main obstacle for enzymatic hydrolysis [30], the effect could tentatively be attributed to improved enzyme accessibility caused by the more severe pretreatment producing a larger surface area by altering the physical shape of the pretreated material, i.e. by creating a more favorable topology.

## Conclusions

Seven fractions of Scots pine were compared with respect to chemical composition and susceptibility to analytical-scale pretreatment and enzymatic saccharification. Chemical analysis showed that the fractions differed significantly as exemplified by the relatively high content of extractives of the knotwood fraction and the relatively high content of ash in the bark fraction. The investigation showed that the different fractions were relatively similar with regard to susceptibility to pretreatment and enzymatic saccharification, which is somewhat unexpected considering the variation between the fractions. The most obvious difference with regard to enzymatic saccharification was that without pretreatment the bark fraction was more susceptible than the other fractions. That different fractions exhibit similar susceptibility facilitates the utilization of forestry residues from Scots pine for bioconversion to fuels and chemicals.

## Methods

### Lignocellulosic material

Different fractions of Scots pine (*Pinus sylvestris*) were obtained from the lower parts (0–16 m height) of six trees with an age between 22 and 145 years, a height between 12.0 and 23.5 m, and a diameter at 1.3 m height between 12 and 35 cm. Each steam was separated into five fractions with a chain saw. The xylem wood (excluding bark and phloem wood) was divided into sapwood and heartwood. These fractions were further divided into juvenile and mature wood, which resulted in four fractions. The juvenile wood (inner part) was separated from the mature wood (outer part) at the 20th annual ring from the pitch. The heartwood and the sapwood were separated visually by their different coloration. The top parts of the pine stems (including bark) were collected as a separate fraction using the chain saw. The five resulting fractions were then chipped in a chipper (Edsbyhuggen 250H, Woxnadalens Energi AB, Edsbyn, Sweden). From the chips containing the top parts, two additional fractions, called knotwood (which had some juvenile sapwood attached) and bark (containing also phloem tissue and some attached wood) were obtained by visual sorting after drying. The juvenile sapwood fraction was used as reference in comparisons between the different fractions. The seven fractions and some of their properties have previously been described by Lestander et al. [8].

### Milling and sieving

The pine fractions were first milled to a particle size of 1 mm [8], but were then milled once more using a Foss Tecator Cyclotech 1093 sample mill equipped with a 0.5 mm ring sieve. The milled material was then sieved in a Retsch Analytical sieve shaker AS 200 (Retsch, Haan, Germany) and the material obtained between 100 and 500 μm sieves was collected for each pine fraction.

### Moisture content

The moisture content of each fraction obtained after milling and sieving was determined (four replicates) using a HG63 Halogen Moisture Analyzer (Mettler-Toledo, Greifensee, Switzerland).

### Determination of the chemical composition of the pine fractions

The determination of extractives was performed as described by Lestander et al. [8] by extraction during 1 h using a Soxhlet system (Büchi Extraction System B-811, Büchi, Flawil, Switzerland) and 200 mL of a 9:1 mixture of petroleum ether (Petroleum Benzene, Merck, Darmstadt, Germany) and acetone for extraction of 3 g of wood. The extracted samples were then dried in air at room temperature for about 16 h until the weight of the samples was stable. The weight of the extracts was measured using a balance (Mettler Toledo XS204) and expressed as percentage of dry substance. For determination of carbohydrates and lignin, the pine fractions were first extracted, to achieve a more accurate estimation. The extraction procedure used was the same as described above, but with the exception that 15 cycles (1–1.5 h) were used in the extraction step instead of a specific extraction time (1 h). The contents of structural carbohydrates (arabinan, galactan, glucan, mannan, and xylan) and lignin (acid soluble and acid insoluble) of extracted wood and bark fractions were determined essentially using a procedure, NREL/TP-510-42618, described by Sluiter et al. [31]. Apart from using a different extraction method, the procedure used also differed with regard to the method used for determination of the concentration of monosaccharides generated by the hydrolysis. The concentrations of the monosaccharides were determined using High-Performance Anion-Exchange Chromatography (HPAEC) as described in the section “Analysis of hydrolysates” below. The determination of the ash content in the different pine fractions was performed using the Swedish Standard method SS-EN 14775.

### Pyrolysis - gas chromatography/mass spectrometry (Py-GC/MS) analysis

The seven pine fractions were analyzed using Py-GC/MS at the Cell Wall and Carbohydrate Analytical Facility of the Umeå Plant Science Center (UPSC) (Umeå, Sweden). The instrument and the procedures used have been described previously [32]. The main objective of the Py-GC/MS analysis was to determine the syringyl-guaiacyl ratio (the S/G ratio) of the lignin of the different pine fractions.

### Dilute-acid pretreatment

Enzymatic digestion of cellulose was performed with and without pretreatment of the pine fractions. The pretreatment was performed with dilute sulfuric acid using an advanced single-mode microwave instrument with stirring to achieve a more uniform temperature distribution in the reaction vessel.

The total mass of the reaction mixture was always 1000 mg. The amount of milled and sieved biomass in each reaction was 50 mg dry weight [5% (w/w)]. The pretreatment reactions were carried out using an Intiator 2.0 EXP instrument (Biotage, Uppsala, Sweden). The reaction vessels were glass vials (0.5-2 mL reaction size, Biotage) equipped with small (10 mm diameter) magnetic stirring bars (Biotage). Three different combinations of temperature, treatment time, and sulfuric acid concentration were used resulting in mild, medium and severe pretreatment conditions (Table 2). The pretreatment conditions can be described in terms of the resulting Combined Severity (CS) [33,34], which ranged between 2.7 and 3.3 (Table 2). For each pine fraction and pretreatment condition, reactions were performed in triplicates.

After pretreatment, the reaction mixture was transferred to pre-weighed 2-mL Sarstedt micro-centrifuge tubes using a 1-mL pipette tip with the end cut off to facilitate quantitative transfer of the content. The pretreatment reaction mixture was centrifuged for 15 min at 14,100 *g* (14,500 rpm) with a MiniSpin Plus centrifuge (Eppendorf, Hamburg, Germany). The solid phase and the liquid phase (referred to as the pretreatment liquid) were then separated. After separation, the pretreatment liquid was stored at -20°C awaiting analysis. The solid phase was washed two times with one mL Milli-Q water (Millipore, Billerica, MA) and one time with one mL sodium citrate buffer (50 mM citrate, pH 5.2). The centrifugation after each washing step was performed as described previously. The pH was checked with a pH indicator and, if necessary, a fourth washing step was performed (with the citrate buffer) so that the final pH was around 5.

### Enzymatic hydrolysis

Enzymatic hydrolysis was carried out in parallell in analytical-scale 2-mL Sarstedt microcentrifuge tubes. The total mass of the reaction mixtures was 1000 mg. For each pine fraction and pretreatment condition, three replicates were processed. The lignocellulosic material in each reaction mixture consisted of 50 mg (dry weight) milled and sieved wood or bark, or, alternatively, the remaining solid fraction after pretreatment of 50 mg (dry weight) milled and sieved wood or bark. An enzyme cocktail consisting of 50 mg of a 1:1 mixture of Celluclast 1.5 L (a cellulase-rich liquid enzyme preparation from *Trichoderma reesei* ATCC 26921) and Novozyme 188 (a cellobiase-rich liquid enzyme preparation from *Aspergillus niger*) was added to each reaction mixture. The enzyme preparations were obtained from Sigma-Aldrich (St. Louis, MO, USA). Celluclast 1.5 L had a stated activity of 700 endoglucanase units (EGU)/g, while Novozyme 188 had a stated activity of 250 cellobiase units (CBU)/g. A sodium citrate buffer (50 mM citrate, pH 5.2) was added so that the final weight of the reaction mixture was 1000 mg. The tubes were incubated at 45°C for 72 h in an orbital shaker (Ecotron incubator shaker, Infors, Bottmingen, Swizerland) set at 170 rpm. The tubes were placed horizontally and were fastened with tape so that the screw caps were facing the same side of the incubator. Samples (10 μL) were withdrawn from the reaction mixtures after 0, 4, 48 and 72 h of incubation. At the end of the incubation, the tubes were centrifuged at 14,100 *g* (14,500 rpm) for 5 min and the supernatants were collected, stored at -20°C, and were then used for sugar analysis. The pellets, presumably consisting mostly of lignin and some remaining cellulose, were also collected and stored at -20°C.

### Analysis of hydrolysates

The glucose concentrations in centrifuged samples taken after four h of incubation of enzymatic reaction mixtures were estimated using a glucometer (Accu-Check Aviva, Roche Diagnostics, Basel, Switzerland). Three replicates were analyzed for each pine fraction and pretreatment condition. The result was used to calculate the glucose production rate (GPR) expressed as g glucose L^-1^ h^-1^.

The sugar content of pretreatment liquids and enzymatic hydrolysates (after 72 h reaction time) was analyzed by using HPAEC. Separation and analysis of monosaccharides was performed using an ICS-5000 system equipped with a CarboPac PA20 guard column (3 × 30 mm), a CarboPac PA20 analytical column (3 × 150 mm), and an electrochemical detector (all parts from Dionex, Sunnyvale, CA). The monosaccharides that were determined included arabinose, galactose, glucose, mannose, and xylose. Prior to analysis, all samples were diluted using ultra-pure water and were filtered through a 0.20 μm syringe-driven filter unit with a nylon membrane (Millipore). The results are reported as mean values of triplicate reactions.

The contents of acetic acid and formic acid were analyzed using HPAEC and the Dionex ICS-5000 system equipped with an IonPac AG15 guard column (4 × 50 mm), an IonPac AS15 analytical column (4 × 250 mm), and a conductivity detector (all parts from Dionex). Prior to analysis, all samples were diluted using ultra-pure water and filtered through a 0.20 μm syringe-driven filter unit with a nylon membrane (Millipore). The reported values are based on analyses of three replicate reactions.

## Competing interests

The authors declare that they have no competing interests.

## Authors’ contributions

TAL and LJJ conceived and designed the study. The main part of the experimental work was carried out by MN under the supervision of SW. MN and LJJ wrote the manuscript, which was revised by SW and TAL. All authors read and approved the final manuscript.

## References

[B1] Sims REH Mabee W Saddler JN Taylor M An overview of second generation biofuel technologies Bioresour Technol 2010 101 1570 1580 10.1016/j.biortech.2009.11.046 19963372

[B2] Pu Y Kosa M Kalluri UC Tuskan GA Ragauskas AJ Challenges of the utilization of wood polymers: how can they be overcome? Appl Microbiol Biotechnol 2011 91 1525 1536 10.1007/s00253-011-3350-z 21796383

[B3] Skogsstyrelsen Skogsstatistisk Årsbok 2012 Jönköping

[B4] Mabee WE Gregg DJ Arato C Berlin A Bura R Gilkes N Mirochnik O Pan X Pye EK Saddler JN Updates on softwood-to-ethanol process development Appl Biochem Biotechnol 2006 129–132 55 70 10.1385/abab:129:1:55 16915631

[B5] Galbe M Zacchi G Pretreatment of lignocellulosic materials for efficient bioethanol production Adv Biochem Eng Biotechnol 2007 108 41 65 17646946 10.1007/10_2007_070

[B6] Saddler JN Bioconverison of Forest and Agricultural Plant Residues 1993 Wallingford: CAB International

[B7] Sjöström E Wood Chemistry: Fundamentals and Applications 1993 2 San Diego: Academic Press

[B8] Lestander TA Geladi P Larsson SH Thyrel M Near infrared image analysis for online identification and separation of wood chips with elevated levels of extractives J Near Infrared Spec 2012 20 591 599 10.1255/jnirs.992

[B9] Bertaud F Holmbom B Chemical composition of earlywood and latewood in Norway spruce heartwood, sapwood and transition zone wood Wood Sci Technol 2004 38 245 256

[B10] Burkhardt S Kumar L Chandra R Saddler J How effective are traditional methods of compositional analysis in providing an accurate material balance for a range of softwood derived residues? Biotechnol Biofuels 2013 6 90 10.1186/1754-6834-6-90 23800175 PMC3704954

[B11] Willför SM Ahotupa MO Hemming JE Reunanen MH Eklund PC Sjöholm RE Eckerman CS Pohjamo SP Holmbom BR Antioxidant activity of knotwood extractives and phenolic compounds of selected tree species Agric Food Chem 2003 51 7600 7606 10.1021/jf030445h 14664514

[B12] Lestander TA Lundström A Finell M Assessment of biomass functions for calculating bark proportions and ash contents of refined biomass fuels derived from major boreal tree species Can J For Res 2012 42 59 66 10.1139/x11-144

[B13] Tao G Lestander TA Geladi P Xiong S Biomass properties in association with plant species and assortments I: A synthesis based on literature data of energy properties Renew Sust Energ Rev 2012 16 3481 3506 10.1016/j.rser.2012.02.039

[B14] Kenney KL Smith WA Gresham GL Westover TL Understanding biomass feedstock variability Biofuels 2013 4 111 127 10.4155/bfs.12.83

[B15] Fengel D Wegener G Wood: Chemistry, Ultrastucture, Reactions 1983 Berlin: Walter de Gruyter

[B16] Glasser WG Glasser HR The evaluation of lignin’s chemical structure by experimental and computer simulation techniques Pap Puu 1981 63 71 74

[B17] Davison BH Sadie RD Tuskan GA Davis DF Nghiem NP Variation of S/G ratio and lignin content in a *Populus* family influences the release of xylose by dilute acid hydrolysis Appl Biochem Biotechnol 2006 129–132 427 435 10.1385/abab:130:1:427 16915659

[B18] Studer MH DeMartini JD Davis MF Sykes RW Davison B Keller M Tuskan GA Wyman CE Lignin content in natural *Populus* variants affects sugar release Proc Natl Acad Sci USA 2011 108 6300 6305 10.1073/pnas.1009252108 21444820 PMC3076829

[B19] Jönsson LJ Alriksson B Nilvebrant N-O Bioconversion of lignocellulose: inhibitors and detoxification Biotechnol Biofuels 2013 6 16 10.1186/1754-6834-6-16 23356676 PMC3574029

[B20] Larsson S Palmqvist E Hahn-Hägerdal B Tengborg C Stenberg K Zacchi G Nilvebrant N-O The generation of fermentation inhibitors during dilute acid hydrolysis of softwood Enzyme Microb Tech 1999 24 151 159 10.1016/S0141-0229(98)00101-X

[B21] Kim KH Tucker M Nguyen Q Conversion of bark-rich biomass mixture into fermentable sugar by two-stage dilute acid-catalyzed hydrolysis Bioresour Technol 2005 96 1249 1255 10.1016/j.biortech.2004.10.017 15734312

[B22] Boussaid A-L Esteghlalian AR Gregg DJ Lee KH Saddler JN Steam pretreatment of douglas-fir wood chips: Can conditions for optimum hemicellulose recovery still provide adequate access for efficient enzymatic hydrolysis? Appl Biochem Biotechnol 2000 84–86 693 705 10.1385/abab:84-86:1-9:693 10849828

[B23] Brownell HH Saddler JN Steam-explosion pretreatment for enzymatic hydrolysis Biotechnol 1984 14 55 68

[B24] Schwald W Brownell HH Saddler JN Enzymatic hydrolysis of steam-treated aspen wood: influence of partial hemicellulosc and lignin removal prior to treatment J Wood Chem Technol 1988 8 543 560 10.1080/02773818808070700

[B25] Jakobsons J Hortung B Erins P Sundquist J Characterization of alkali soluble fraction of steam exploded birch wood Holzforschung 1995 49 51 59 10.1515/hfsg.1995.49.1.51

[B26] Sannigrahi P Dong KH Seokwon J Ragauskas A Pseudo-lignin and pretreatment chemistry Energy Environ Sci 2011 4 1306 1310 10.1039/c0ee00378f

[B27] Kumar R Hu F Sannigrahi P Jung S Ragauskas AJ Wyman CE Carbohydrate derived-pseudo-lignin can retard cellulose biological conversion Biotechnol Bioeng 2013 110 737 753 10.1002/bit.24744 23042575

[B28] Ucar G Fengel D Characterization of the acid pretreatment for the enzymatic-hydrolysis of wood Holzforschung 1988 42 141 148 10.1515/hfsg.1988.42.3.141

[B29] Ramos LP Nazhad MM Saddler JN Effect of enzymatic hydrolysis on the morphology and fine structure of pretreated cellulosic residues Enzyme Microb Technol 1993 15 821 831 10.1016/0141-0229(93)90093-H

[B30] Arantes V Saddler JN Cellulose accessibility limits the effectiveness of minimum cellulase loading on the efficient hydrolysis of pretreated lignocellulosic substrates Biotechnol Biofuels 2011 4 3 10.1186/1754-6834-4-3 21310050 PMC3042927

[B31] Sluiter A Hames B Ruiz R Scarlata C Sluiter J Templeton D Crocker D Determination of structural carbohydrates and lignin in biomass 2008 Golden, Colorado: National Renewable Energy Laboratory NREL/TP-510-42-618

[B32] Gerber L Eliasson M Trygg J Moritz T Sundberg B Multivariate curve resolution provides a high-throughput data processing pipeline for pyrolysis-gas chromatography/mass spectrometry J Anal Appl Pyrol 2012 95 95 100

[B33] Overend RP Chornet E Fractionation of lignocellulosics by steam-aqueous pretreatments Phil Trans R Soc London, Series A 1987 321 523 536 10.1098/rsta.1987.0029

[B34] Chum HL Johnson DK Black SK Overend RP Pretreatment-catalyst effects and the combined severity parameter Appl Biochem Biotechnol 1990 24/25 1 4 10.1007/BF02920229

